# Gone fishin’… for distinct patterns of belief-updating in late-life worry and rumination

**DOI:** 10.3389/fpsyt.2026.1824838

**Published:** 2026-07-15

**Authors:** Angela M. Ianni, Vanessa M. Brown, Andrea M. Weinstein, Mingqian Li, Carmen Andreescu, Andrew R. Gerlach

**Affiliations:** 1 Department of Psychiatry, University of Pittsburgh, Pittsburgh, PA, United States; 2 Department of Psychology, Emory University, Atlanta, GA, United States; 3 Department of Neuroscience, University of Pittsburgh, Pittsburgh, PA, United States; 4 Department of Bioengineering, University of Pittsburgh, Pittsburgh, PA, United States

**Keywords:** bayesian model, belief-updating, computational psychiatry, repetitive negative thinking, rumination, worry

## Abstract

**Introduction:**

Worry and rumination are two common forms of repetitive negative thinking encountered as core symptoms of anxiety and depression. They are difficult to treat and increase the risk of relapse for remitted depressive and anxiety disorders, especially in late-life. We propose that dysfunctional belief-updating is a fundamental cognitive mechanism underlying repetitive negative thinking and use a Bayesian model to test whether distinct components of belief updating are differentially associated with worry and rumination.

**Methods:**

We recruited 83 older participants (age≥50) dimensionally for worry and rumination to perform a belief-updating task and undergo neuropsychological testing. We extracted three parameters from a Bayesian model of belief-updating: performance compared to the Bayesian optimal model, emphasis on initial information (prior weight), and relative weighting of new evidence compared to current beliefs (update strength). These parameters were tested for associations with worry and rumination severity, and four neuropsychological domains were tested as moderators (attention, visuospatial, working memory maintenance, and executive function).

**Results:**

Worry severity was uniquely associated with lower prior weight, while rumination was associated with low update strength. Neither worry nor rumination were associated with performance. None of the neuropsychological domains moderated these relationships.

**Conclusion:**

Worry and rumination are associated with unique alterations in belief-updating but not overall performance. Low prior weight in worry may inflate perceived uncertainty, while low update strength in rumination—specifically reflective pondering—is consistent with slower incorporation of new information. Both biases may contribute to the distinct phenomenological profiles of worry and rumination and inform specific therapeutic targets for these symptoms.

## Introduction

Worry and rumination are common symptoms across a wide range of psychiatric disorders (1) and are associated with subjective impairments in cognition and physical health (2). In depression and anxiety, they are also linked to poor treatment response (3) and recurrence of symptoms even after successful treatment (4, 5). Worry and rumination are also notoriously difficult to treat (3), particularly in older adults (6), where the repetitive cycles are often entrenched as habit. This is especially concerning given that anxiety and depression—disorders in which worry and rumination are cardinal features—carry substantial consequences in later life, including elevated cardiovascular risk (7–10) and increased likelihood of dementia (11–13). Anxiety in particular shows a second peak of incidence around age 50 (14, 15), meaning that later life represents a period of heightened vulnerability rather than relief from these concerns. Critically, our group has found that worry and rumination specifically are associated with accelerated brain aging above and beyond broader diagnostic categories (16). This suggests that the transdiagnostic symptoms of worry and rumination may be particularly pernicious in aging populations and represent important targets for mechanistic studies.

Worry is a core component of anxiety disorders commonly defined as “a chain of thoughts and images, negatively affect-laden and relatively uncontrollable” (17) that is typically future-directed (18). Rumination, on the other hand, is defined as “the tendency to repetitively analyze one’s problems, concerns, and feelings of distress without taking action to make positive changes” (19) and is more associated with depression. Specifically, rumination is posited to play a key role in the maintenance of depressive symptoms (20) and is typically more past/present focused, though the temporal orientation is less distinct than with worry (21). Further, rumination has been decomposed into adaptive and maladaptive subtypes: reflective pondering and brooding, respectively (22, 23). Traditionally, worry has been studied in the context of anxiety and rumination in the context of depression, and the content of the thoughts has been the focus. Recently there has been a push to unify worry, rumination, and related processes under the transdiagnostic umbrella of repetitive negative thinking (RNT) (24, 25). Self-report data has reliably identified a core RNT component common to both worry and rumination (25). Under this framework, the process of RNT takes primacy over the content, which has important implications for psychotherapeutic approaches (25). RNT is thought to arise from the maladaptive extension of otherwise healthy, adaptive cognitive mechanisms (17, 26). For example, planning for future contingencies is a part of everyday life. Thus, a certain amount of worry is beneficial for survival, but too much worry becomes problematic. Similarly, reflection on past events allows us to learn from experience and update beliefs for the future, but perseverative brooding laced with negative emotionality define unhealthy rumination. The adaptive goals of planning and reflection require successful updating of current beliefs to arrive at productive conclusions. For example, imagine you texted your partner and did not receive a response. You may start to worry that they’re mad at you and start wracking your brain for transgressions that may have upset them–*I shouldn’t have left those dirty dishes in the sink–*and continue to catastrophize the situation, growing more and more concerned. Or you could remember that they have a meeting today and may be busy or that they wished you a good day as you left the house. These thoughts can be conceptualized as evidence used in the belief-updating process. Deficits in the belief-updating process may preclude such successful resolution, warping the adaptive process of planning and reflection into RNT. Therefore, we hypothesize that dysfunctional belief-updating may be one of the fundamental cognitive mechanisms underlying RNT.

Belief-updating is the process of adjusting conceptions of the world as new information is gathered (27). Importantly, belief-updating can be captured with computational models of behavior, which allows for a more objective quantification of behavior than self-reports and offers a tractable bridge between the brain and behavior (28, 29). Anxiety and depression have been studied extensively in the context of computational models (30, 31). Maladaptive learning and altered processing of uncertainty have emerged as key features of anxiety disorders, spanning fear-based threat learning (32, 33) and distress-related intolerance of uncertainty (34). The research in depression has been less decisive (35), with many studies focusing on specific symptoms or subtypes of depression, most notably anhedonia owing to its clear theoretical link to reward-based computational approaches (36). Notably, many computational studies of depression and anxiety identify reliable differences in model-derived behavioral parameters (35, 37) without observing consistent differences in overall task performance relative to healthy controls (38, 39). These latent parameters may capture more subtle alterations in underlying cognitive processes that do not always manifest as degraded overall performance, especially on tasks that tend to engage more “cold-cognition” (i.e., emotion-independent) than affective circuitry key to anxiety and depression (40). Given the remarkable symptomatic heterogeneity encountered at the disorder level (41), diagnostic categories such as anxiety and depression may obscure more specific cognitive mechanisms. Indeed, computational approaches have often made greater progress when focusing on specific symptom dimensions rather than broad diagnoses (e.g., anhedonia as noted above). A similar approach may therefore help clarify how belief-updating processes contribute to transdiagnostic phenomena such as RNT. Further, by disentangling RNT symptoms from specific disorders, we can broaden the findings across a wider range of psychopathology (42).

Belief-updating has been formalized within a Bayesian framework, which quantifies how prior beliefs and sensory information are weighted to form inferences (43). In psychiatry, Bayesian models have been widely applied (44), particularly in schizophrenia, where aberrant belief updating has been linked to hallucinations and delusions (45–47). Mathematically, belief-updating can be concisely described with Bayes’ formula: updated beliefs (posterior) reflect a precision-weighted combination of previous beliefs (prior) and evidence from new observations (likelihood). However, this relies on multiple component cognitive processes. Effective belief-updating requires the allocation of attention to incoming evidence, accurate perception of stimulus features, maintenance and manipulation of probabilistic information in working memory, and executive control to integrate new information with existing beliefs. These processes are particularly relevant in older adults, since the normal aging process is characterized by substantial inter-individual variability in cognitive function and known declines in processing speed, attention, memory, and executive function (48), particularly in the presence of heightened RNT (49). Age-related differences in attention, visuospatial processing, working memory maintenance, and executive function have each been shown to influence learning, decision-making, and belief formation independently of psychiatric symptoms (50, 51).

In this study, we examined whether worry and rumination were associated with aberrations in belief-updating in a sample of older adults enriched for RNT. We focused on three belief-updating parameters: 1) similarity to Bayesian optimal performance, 2) prior weight (emphasis placed on initial information), and 3) update strength (relative weighting of new evidence compared to current beliefs). Based on previous studies in depression and anxiety that broadly found performance to be intact across reward-learning tasks (37), we did not expect worry or rumination to be associated with overall performance compared to the optimal Bayesian model. Given the well-established deficits in learning about uncertainty and maladaptive use of uncertainty in anxiety (32, 33) and the more obviously stochastic nature of observations compared to prior information in our task, we expected worry severity to be associated with greater prior weight. Finally, we expected rumination to be associated with lower update strength, reflecting reduced belief revision and difficulty disengaging from past events (20, 52), and specifically of discarding irrelevant information from working memory (53–55). To distinguish belief-updating alterations specific to RNT from more general cognitive processes, we also examined how performance across the cognitive domains of attention, visuospatial acuity, working memory, and executive function related to belief-updating parameters and whether they mediated observed associations with RNT. This approach allows us to test whether RNT-related differences in belief-updating reflect symptom-specific computational alterations rather than secondary consequences of broader cognitive aging.

## Methods

### Participants

We recruited 117 participants aged 50 and older through the Rumination, Anxiety, and Worry (RAW) Brain study (R01 MH108509) examining the effects of rumination, anxiety and worry on aging and dementia risk. RAW is a longitudinal observational study with visits 2 years apart including demographic, clinical, behavioral, plasma biomarkers, and MRI data collection; the data presented in this manuscript is from the baseline visit only. Participants were recruited through Pitt+Me (a web-based University of Pittsburgh research study recruitment resource), BuildClinical (a data-driven platform that helps academic researchers recruit participants for research studies more efficiently using social media, software, and machine learning), in-person referrals, and flyers. Recruitment was stratified by age and RAW scores (see definition in Assessments) to ensure even representation across these domains. The minimum age of 50 corresponds to the second peak of generalized anxiety disorder (GAD) onset in later-life (14) (the other peak is in adolescence). Given the focus of this study on anxiety phenotypes, participants with and without mood and anxiety disorders were included. This study was approved by the University of Pittsburgh Institutional Review Board and all participants provided written informed consent prior to participation in the study.

Inclusion criteria for the RAW study were age 50 and older and 1) healthy and non-anxious; 2) anxiety symptoms without DSM-5 diagnoses; or 3) DSM-5 diagnosis of GAD, other anxiety disorders (e.g., panic disorder, social phobia), and/or mood disorders such as major depressive disorder (MDD), persistent depressive disorder, or unspecified depressive disorder. Diagnoses were assessed with the Mini International Neuropsychiatric Interview (56) (MINI). The telephone Montreal Cognitive Assessment (T-MoCA) (57) was used to screen for cognitive impairment. Exclusion criteria were: University of California, San Deigo Brief Assessment of Capacity to Consent (UBACC) score < 14.5, indicating an inability to provide direct consent; diagnosis of autism spectrum disorders, intellectual development disorder, or any form of psychosis or bipolar disorder; diagnosis of major neurocognitive disorder (e.g. dementia); MoCA score < 24 (or T-MoCA score < 13); suicide risk as determined as a score above 5 on the MINI suicide module; use of antidepressant medication within the past two weeks (six weeks for fluoxetine) unless currently high RAW (must have been established on the medication for 12 weeks or longer); history of alcohol abuse within the last six months; history of drug abuse within the past 12 months with the exception of marijuana; use of high doses of benzodiazepines; uncorrected vision problems; below 6th grade reading level; clinical diagnosis of stroke, multiple sclerosis, vasculitis, or significant head trauma; untreated or unstable medical conditions affecting variables of interest; contraindications for MRI; positive pregnancy test; inability or unwillingness to taper off of particular medications; positive urine drug screen except marijuana and/or due to a prescribed medication.

### Assessments

All participants completed a battery of psychometric and cognitive assessments at the baseline visit. Rumination was assessed with the Response Styles Questionnaire – Rumination subscale (20, 58) (RSQ); worry was assessed with the Penn State Worry Questionnaire (59) (PSWQ); anxiety was assessed with the Hamilton Anxiety Rating Scale (60) (HARS); and depression was assessed with the Hamilton Depression Rating Scale (61) (HDRS). The RSQ can be further stratified into brooding and reflective pondering subtypes (22, 23). RSQ, HARS, and PSWQ were used to calculate RAW scores for recruitment stratification with cutoffs of RSQ ≥ 50, HARS ≥ 17, and PSWQ ≥ 55 defining high RAW. Cognitive assessments included the Delis–Kaplan Executive Function System (62) (D-KEFS) Color Word Interference Test and Trail Making Test and the Repeatable Battery for the Assessment of Neuropsychological Status (63) (RBANS).

Cognitive measures were selected, normed for age, and averaged to create index scores representing visuospatial, working memory maintenance, executive functioning, and attention domains. The RBANS Visuospatial/Construction index, derived from Line Orientation and modified Figure Copy raw scores, assesses basic visuospatial perception and design reproduction. The Working Memory Maintenance Index captures encoding and short-term retention and included RBANS List Learning total score, Story Memory Learning total score, and Digit Span raw score. The Executive Functioning Index comprised of the D-KEFS Trail Making Test Condition 4 vs. Condition 5 scaled score (Number-Letter Switching, corrected for motor speed, assessing cognitive flexibility), and the Color-Word Interference Test Condition 3 (inhibitory control) and Condition 4 (inhibition/switching) weighted combined scaled scores. The Attention Index included RBANS Digit Span (auditory attention) and Coding (timed symbol-number matching) raw scores.

### Belief-updating task

**Figure 1:**
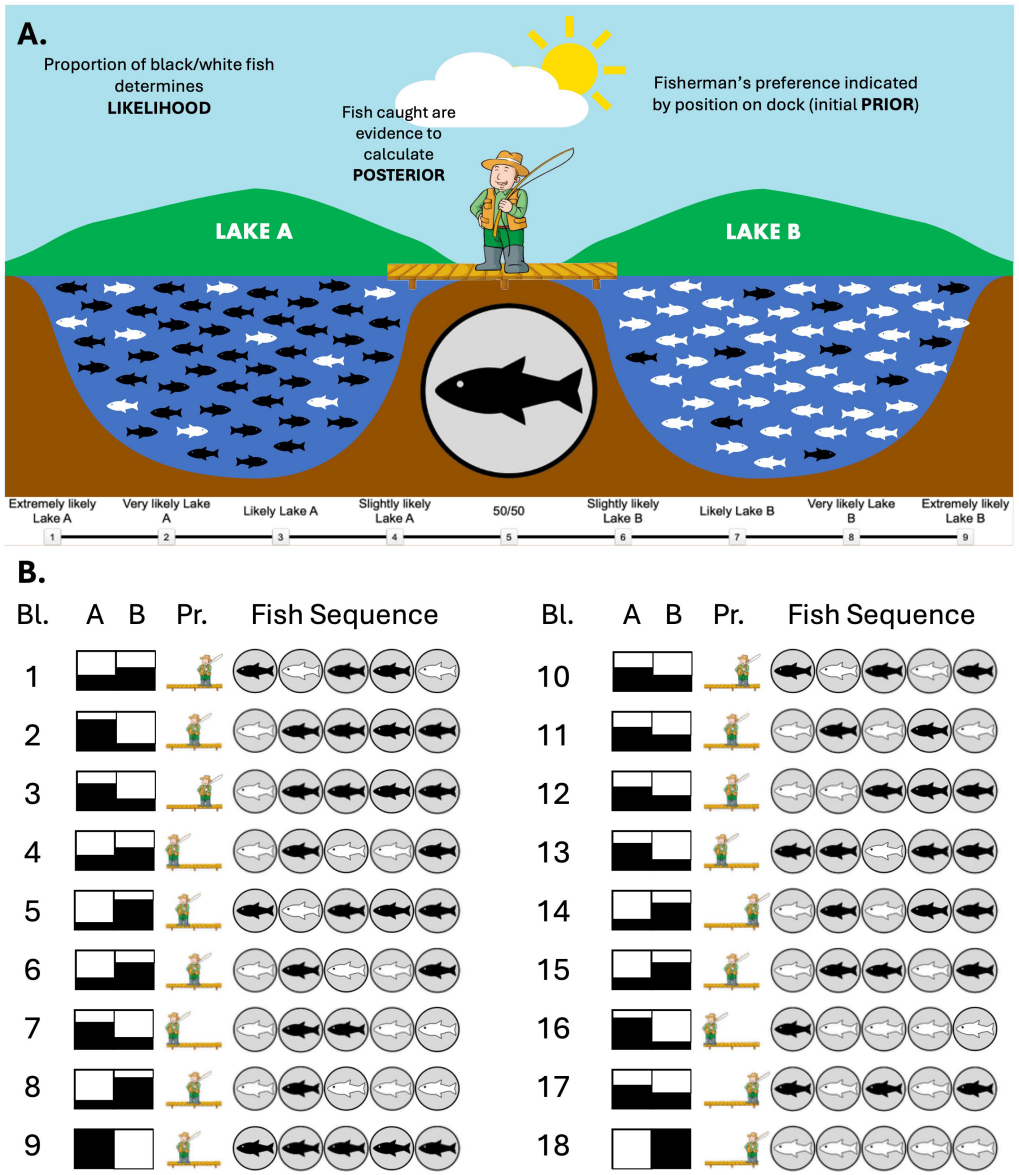
*Fish task overview*. **(A)** For each block, participants are shown two lakes (Lake A and Lake B) with opposing proportions of black and white fish and the fisherman’s position on the dock that expresses his preference for the two lakes (initial prior). Participants are then shown a series of 5 fish caught from one of the lakes and asked to rate the probability that the fisherman is fishing in Lake A or Lake B using a 9-point Likert scale 6 times for each block: once based solely on the dock position and once after each fish is caught. In this example, the fisherman is standing in the middle of the dock and thus expressing no preference for either lake, and we would expect participants to provide an initial probability rating of 5 (50/50). After catching the black fish shown, we would expect participants to shift their rating toward Lake A **(B)**. Task parameters for each block (Bl.): proportion of fish in Lake A **(A)**, proportion of fish in Lake B **(B)**, the fisherman’s position on the dock (prior, Pr.), and the sequence of five fish caught in that block. Note that the lakes in block 9 and 18 contain all white or all black fish.

Belief-updating was assessed with a modified version of the Fish task (64), which itself is a variant of the Beads task (65) that replaces a binary decision with a probability rating. This task was selected because the participant-provided probability ratings provide direct access to the belief-updating process without the need to infer latent beliefs. Further, the relatively simple task design allows for repeated sampling of participant behavior over a variety of task conditions to produce reliable parameter estimates (66). In the Fish task, participants must infer which of two lakes a fisherman is fishing in. The two lakes (Lake A and Lake B) contain opposing proportions of black and white fish. This version of the task incorporates an explicit prior–the fisherman’s preference for fishing in the two lakes is expressed by his position on the dock. Participants are shown a series of 5 random fish caught from one of the lakes. After seeing the fisherman’s position on the dock and after each fish is caught, participants are asked to rate the how likely it is that the fisherman is fishing in Lake A or Lake B (posterior) using a 9-point Likert scale. Likelihood is a function of the proportion of fish in the lakes and the sequence of fish caught. The Fish task was coded using the Inquisit 6 software and administered in-person on a study-provided iPad. An overview of the task is shown in **Figure 1**.

Participants completed 18 blocks of the task with prior preference, proportion of fish in each lake, and sequence of fish caught varying each block. Prior preference was expressed at 5 possible positions for the fisherman on the dock: middle (no preference), halfway toward Lake A or B (moderate preference), and all the way toward Lake or B. Proportion of black/white fish varied from 60%/40% to 80%/20% in increments of 10%, with the opposing lake always containing the opposite proportion of black/white fish (i.e., 40%/60% to 20%/80%). The sequences of fish were preselected to even coverages of the task parameter space and include scenarios that are likely to reveal individual differences. For example, if the fisherman expresses a strong preference for Lake A and then catches 4 of the dominant color fish from Lake A (i.e., the color of fish with the higher proportion in Lake A) followed by one non-dominant fish, this is unlikely to yield significant variability among participant response, but the reverse ordering (one non-dominant fish followed by 4 dominant fish) would require participants to reconsider their prior for Lake A and then reverse course back to Lake A, which is likely to yield significant variability in response. Blocks 9 and 18 were catch blocks used to assess participants’ comprehension of the task; only white fish appeared in one lake and only black fish appeared in the other lake, thus trivializing the likelihood calculation. Thus, 16 blocks with 5 trials each (80 trials total) were used in all analyses, while blocks 9 and 18 were only included in analyses of prior weight. Participants were excluded if they exhibited 1) “stagnant” behavior (did not adjust their ratings on at least 50 of the 80 trials) suggesting they did not engage in the task, 2) “swingy” behavior (switched which lake they rated as most probable at least 50% more than the Bayesian optimal model) suggesting that the participant did not understand that all of the fish caught within a block came from the same lake; or 3) displayed other behavior that was not analyzable (explained in results). In general, we employed liberal thresholds to allow for as much data inclusion as possible and provide example of excluded data in **Figure 1**.

### Parameter extraction

We extracted three parameters of interest from the Fish task: task performance compared to the Bayesian optimal model, prior weight, and update strength. Prior weight captures the amount of confidence participants placed in their rating based solely off the position of the fisherman on the dock and was quantified as Equation 1.

(1) $$\begin{array}{c} w_{prior}=\frac{1}{12}\underset{b\notin \text{middle}}{\sum }|r_{b,\;prior}-0.5| \end{array}$$

where b indicates block, b∉middle indicates blocks where the fisherman is anywhere but the middle of the docks (i.e., expressing some preference for one of the lakes), $r_{b,prior}$ is the probability rating (9-point Likert rating divided by 10) for the block b after observing only the fisherman’s position on the dock, and $w_{prior}$ is the average prior weight for a participant. There are 12 blocks where the fisherman is not positioned in the middle of the dock, hence the 1/12 normalizing factor.

Performance was quantified by comparing participants responses to an ideal Bayesian observer, which simply utilizes Bayes’ formula to update beliefs based on prior information and likelihood of observations with Equation 2.

(2) $$\begin{array}{c} p(A|B)=\frac{p(A)p(B|A)}{p(B)}\text{ or posterior}=\frac{\text{prior }\times \;\text{likelihood}}{\text{marginal likelihood}} \end{array}$$

For the Fish task, the likelihood can be calculated based on the proportion of fish in each lake and the sequences of fish caught, as shown in Equation 3.

(3) $$\begin{array}{c} p_{b,i}=\frac{1}{1+\frac{\left(1-p_{b,i-1})*[\beta_{b,W}I_{i,W}+(1-\beta_{b,W})(1-I_{i,W})\right]}{p_{b,i-1}*[\alpha_{b,W}I_{i,W}+(1-\alpha_{b,W})(1-I_{i,W})]}} \end{array}$$

where $p_{b,i}$ is the probability that the fisherman is fishing in Lake A for block *b* after observing the $i^{th}$ caught fish ( i∈[1, 2, 3, 4, 5]), $\alpha_{b,W}$ is the proportion of white fish in Lake A in block b, $\beta_{b,W}$ is the proportion of white fish in Lake B in block b ( $\beta_{b,W}=1-\alpha_{b,W}$ by task design), $I_{i,W}$ is 1 if the $i^{th}$ fish is white and 0 if black, and $p_{b,0}=r_{b,prior}$. Note that this final definition means that the first trial uses the use-provided prior rating as the prior since any other quantification of the fisherman’s position on the dock would be arbitrary, while all other trials use the previous trial’s posterior as the prior. Performance compared to the ideal Bayesian observer is then simply calculated as shown in Equation 4.

(4) $$\begin{array}{c} \delta =\frac{1}{80}\underset{b}{\sum }\underset{i}{\sum } \end{array}|r_{b,i}-p_{b,i}|$$

Where $r_{b,i}$ is the probability rating (9-point Likert rating divided by 10) for block b after observing the $i^{th}$ caught fish, $p_{b,i}$ is the ideal Bayesian observed probability as defined in equation (3), and the normalizing factor of 80 is to average over the total number of trials.

Finally, to calculate update strength, we fit a parameterized version of Bayes’ formula that allows for the prior and likelihood to be weighted variably for each participant (47). To do this, we take the logarithm of equation (2), which allows us to rewrite Bayes’ formula as a linear combination of terms, as shown in Equation 5.

(5) $$\begin{array}{c} \log(\text{posterior})=\log(\text{prior})+\log(\text{likelihood})-\log(\text{marginal likelihood}) \end{array}$$

By letting $x_{1}=\log(\text{prior})$, $x_{2}=\log(\text{likelihood})$, and y=log(posterior) and noting that the marginal term can be treated as an intercept, we can rewrite equation (5) as linear regression, as shown in Equation 6.

(6) $$\begin{array}{c} y\;\sim \;\beta_{0}+\beta_{1}x_{1}+\beta_{2}x_{2} \end{array}$$

where $\beta_{1}$ is the inverse prior weight (smaller $\beta_{1}$ means greater prior weight) and $\beta_{2}$ is the inverse likelihood weight (smaller $\beta_{2}$ means greater likelihood weight). Note that this is an inversion of the typical nature of the β coefficients that arises due to the nature of the transformation. Specifically, the prior, likelihood, and posterior are all between 0 and 1, so the log of these values ( $x_{1}$, $x_{2}$, and *y*) are all negative. This means that a larger β makes y more negative, and hence exerts less influence on the posterior. (Alternatively, by reversing the transformation, we note that the β‘s become exponents on the prior and likelihood in Bayes’ formula, with larger exponents having a shrinking effect and smaller exponents having an amplifying effect on values between 0 and 1.) Equation (6) was fit with a mixed effects models with a random intercept for participants and estimated values of $\beta_{1}$ and $\beta_{2}$ for each participant were extracted from the models. Update strength was then quantified as $\beta_{1}-\beta_{2}$ with the interpretation that greater update strength places more emphasis on the recent observation (likelihood) than past observations (prior). This can alternatively be interpreted as a decay term. For interpretation purposes, we note that a perfectly Bayesian process would have $\beta_{1}=\beta_{2}=1\to \beta_{1}-\beta_{2}=0$. Model comparison analyses and parameter recovery confirmed that this model provided the best fit and yielded identifiable parameters (see Supplementary Materials).

### Statistical analysis

We first assessed the overall fit of the Bayesian optimal model to participant data. Model performance was assessed by testing for an association between participant-provided probabilities and the Bayesian optimal probabilities using a multilevel model with a random intercept for participants. We tested if update strength differed from zero using a one-sample *t*-test.

Next, we assessed for effects of clinical and cognitive variables on trial-wise deviation from Bayesian optimal and prior weight using multilevel models with random intercepts for participants. We used linear regression to test for associations between update strength and clinical and cognitive variables. Both worry and rumination were included in each model; sensitivity analyses were run for each model with worry and rumination separately as well. Models with positive results for rumination were subjected to additional sensitivity analyses replacing rumination scores with the brooding and reflective pondering subscales. Likewise, all four cognitive indices (visuospatial, working memory maintenance, executive function, and attention) were included in single models testing cognitive variables. To address skewness, the visuospatial index score was reflected, log-transformed, and reflected again to retain its original direction.

We present results with raw p values but note that all results survive false discovery rate (FDR) correction across the family of 3 tests. Results were unchanged in sensitivity analyses with individual measures in separate models. Age, sex, and years of education were included as covariates in all models, but effects were unchanged in sensitivity analyses without these covariates. As additional sensitivity analyses, we included 1) measures of overall anxiety (HARS) and depression (HDRS) symptoms as covariates in the model and 2) GAD and MDD diagnosis; again, results were not affected. Grubb’s test was used to assess for outliers. *Post-hoc* tests quantified contribution of individual cognitive measures to the behavioral associations. All statistical analysis was performed in R (version 4.5.1). Linear models were fit using ordinary least squares with the standard *lm* function, while linear mixed effects models were fit with the *lmer* function in the *lme4 (*67) package using the Bound Optimization By Quadratic Approximation optimizer. All continuous variables were *z*-scored so that β coefficients represent standardized effects.

## Results

### Demographics, clinical, and cognitive characteristics

Of the 117 participants recruited, 2 participants did not complete the task and 31 were excluded for aberrant behavioral data: 22 were removed for “stagnant” behavior (did not adjust their ratings on more than 50 of the 80 trials; 12 did not adjust their ratings at all) suggesting they did not engage in the task; 8 were removed for “swingy” behavior (switched which lake they rated as most probable at least 50% more than the Bayesian optimal model) suggesting they did not understand that all of the fish caught during a block came from the same lake, and 1 participant was removed for only providing ratings of 1 and 5. See **Figure 1** for examples of excluded participant behavior. Demographics of the 83 participants included in the analysis are summarized in **Table 1**. Unsurprisingly, worry and rumination were highly correlated ( $r=0.58,t_{81}=6.04,p<0.01$), though the variance inflation factor was <2 in all models, suggesting that worry and rumination contained sufficient independent variance.

**Table 1:** *Demographic summary of participants (n=83)*.

Measure	n	%
Sex		
Female	56	67.5%
Male	27	32.5%
Race		
White	77	92.8%
Black	4	4.8%
Asian	1	1.2%
Multiracial	1	1.2%
GAD Diagnosis	17	20.5%
Other Anxiety Disorder Diagnosis*	11	13.3%
MDD Diagnosis	13	15.7%
MDE Diagnosis	46	55.4%
Measure	Mean	St. Dev.
Age	62.5	7.8
Education (years)	17.0	2.5
Worry (PSWQ)	48.2	14.7
Rumination (RSQ)	41.3	11.8
Anxiety (HARS)	11.2	7.6
Depression (HDRS)	7.7	4.6
T-MoCA	20.0	1.5

*Other anxiety disorder diagnoses include social anxiety disorder (n=4), panic disorder (n=7), posttraumatic stress disorder (n=3), and agoraphobia (n=2).

Generalized Anxiety Disorder, GAD; Hamilton Anxiety Rating Scale, HARS; Hamilton Depression Rating Scale, HDRS; Major Depressive Disorder, MDD; Major Depressive Episode, MDE; Penn State Worry Questionnaire, PSWQ; Response Style Questionnaire rumination subscale, RSQ; Telephone Montreal Cognitive Assessment, T-MoCA.

### Model summary

**Figure 2:**
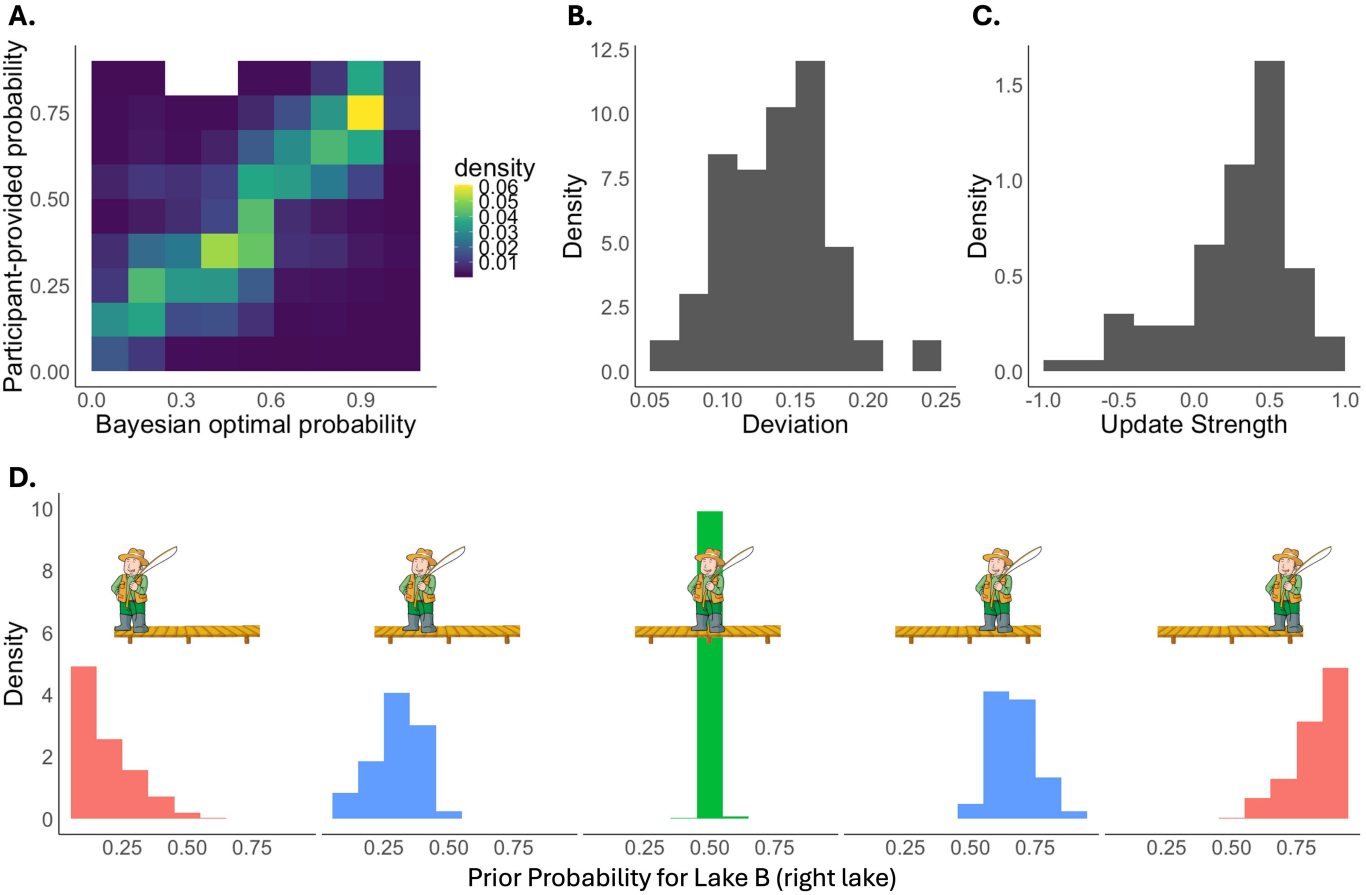
*Model summary*. **(A)** Trial-wise participant-provided probability ratings showed excellent agreement with the Bayesian optimal probabilities. **(B)** Histogram of participant-average deviations. **(C)** Histogram of participant update strength. **(D)** Trial-wise prior probability ratings for Lake B (right lake) as a function of the fisherman’s preference as indicated by his position on the dock.

Overall, participant behavior showed strong agreement with the Bayesian optimal model ( β=0.75,t=92.2,p<0.01; **Figure 2**) with an average difference between the model and participant-provided ratings of 0.14 ± 0.13 (**Figure 2**). Participants were highly logical with their valuation of the prior (**Figure 2**): when the fisherman was in the center of the dock, only 5 of the initial prior ratings differed from 0.5 (1.0%), and only 1 of the initial prior ratings was biased toward the opposite lake indicated by the Fisherman (0.1%). The average update strength was 0.28 ± 0.37, which was significantly greater than the Bayesian optimal value of 0 ( $t_{82}=7.09,p<0.01$; **Figure 2**). One participant with an exceptionally low update strength was identified as an outlier ( G=3.15,p=0.05) and excluded from the update strength analysis.

### Clinical associations with model-based indices

**Figure 3:**
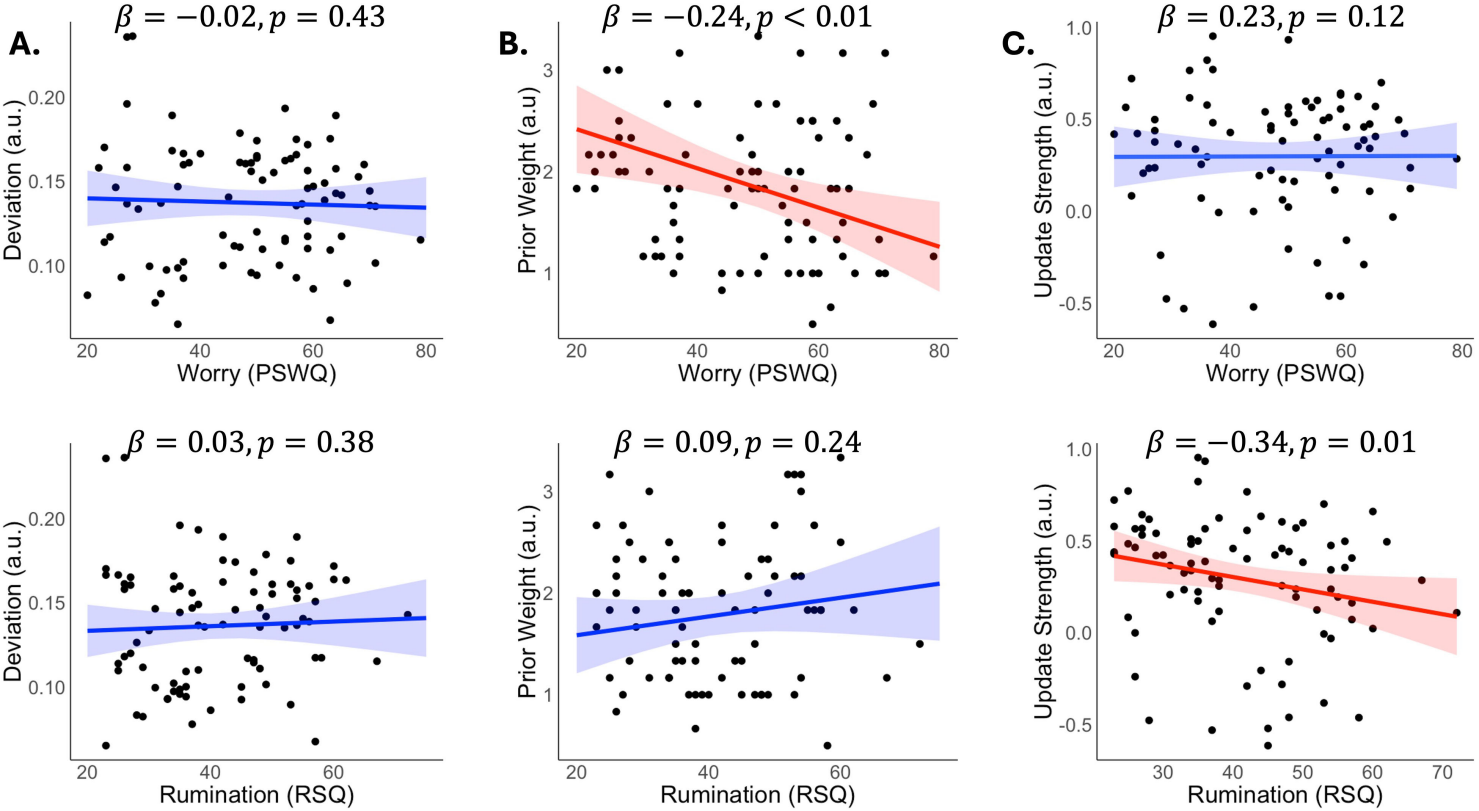
*Clinical associations with belief-updating (significant results shown in red)*. **(A)** Neither worry nor rumination were associated with performance. **(B)** Worry was associated with prior weight, but rumination was not. **(C)** Rumination was associated with update weight, but worry was not. Plots in panels **(A, B)** show participant-average deviation and prior weight, respectively, though actual tests used trial-wise data in multi-level models. Penn State Worry Questionnaire (PSWQ); Response Style Questionnaire rumination subscale (RSQ).

Neither worry nor rumination were associated with performance (worry: β=−0.02,t=−0.79,p=0.43; rumination β=0.03,t=0.87,p=0.38; **Figure 3**; **Table 1**). Worry severity, but not rumination, was inversely associated with prior weight (worry: β=−0.24,t=−2.89,p<0.01; rumination β=0.09,t=1.17,p=0.24; **Figure 3**; **Supplementary Table 2**). Conversely, rumination severity, but not worry, was inversely associated with update strength (worry: β=0.23,t=1.59,p=0.12; rumination =−0.34,t=−2.55,p=0.01; **Figure 3**; **Supplementary Table 3**). Sensitivity analyses revealed that this inverse association with update strength was specific to the reflective pondering component of rumination (**Supplementary Table 4**). Inclusion of overall anxiety and depressive symptoms or anxiety and depression diagnosis did not affect results or reveal independent effects of diagnostic categories (**Supplementary Tables 5**–**10**).

### Cognitive associations with model-based indices

At the compositive score level, greater working memory maintenance was associated with more optimal performance (*β* = −0.14, *t* = −2.23, *p* = 0.03), and update strength was negatively associated with visuospatial processing ( β=−0.36, t=−2.68, p=0.01); see **Figure 2**. Notably, the association between rumination and update strength remained significant after adjusting for visuospatial processing. No significant relationships were observed between prior weight and any cognitive index (all p's>0.25). Additionally, no cognitive indices were associated with worry or rumination or moderated the relationship between worry and prior weight or rumination and update strength.

*Post-hoc* tests examining the effects of individual cognitive measures indicated that the association between working memory maintenance and performance was primarily driven by RBANS List Learning, with a smaller contribution from Digit Span. The association between visuospatial processing and update strength was driven by Line Orientation. Additional *post-hoc* tests of individual cognitive measures indicated that more optimal performance was associated with better Line Orientation scores and higher D-KEFS Condition 4 vs. Condition 5 scaled scores.

## Discussion

To our knowledge, this is the first study to investigate repetitive negative thinking in the context of belief-updating. Using a Bayesian belief-updating task, we demonstrated that older adults were able to perform the task acceptably and showed good overall agreement with the underlying computational model. We found that neither worry nor rumination was associated with overall task performance, though each RNT construct was associated with a unique aberration in the belief-updating process. Specifically, greater worry severity was associated with a lower emphasis placed on the initial task information provided at the beginning of each block (low prior weight), while greater rumination was associated with a slower incorporation of evidence within each block (low update strength). In isolation, these distinct patterns of belief-updating share the common consequence of slowing the belief-updating process, which may be a key mechanism for the repetitive aspect of RNT.

The association between worry severity and lower prior weight suggests that worry is characterized by diminished reliance on pre-existing information when forming beliefs. In the context of our task, this manifested as reduced emphasis on the fisherman’s preference at the beginning of each block, despite this information being stable. This may reflect inflated subjective uncertainty about the environment, leading individuals to discount prior information and require additional evidence before committing to a belief. This pattern aligns with clinical descriptions of worry as a future-oriented process marked by maladaptive use of uncertainty and persistent information seeking (17, 18, 33), though it remains unclear how broadly this pattern generalizes across contexts. Notably, the direction of this effect is opposed to our hypothesis, which conflated maladaptive use of uncertainty with uncertainty aversion.

In contrast–and consistent with our hypothesis–rumination severity was selectively associated with reduced update strength, reflecting slower incorporation of new evidence as it accrued over time. Sensitivity analyses showed that this association was specific to the reflective pondering component of rumination. Across the whole sample, update strength was significantly greater than zero, indicating a relatively greater weighting of current observations compared to prior beliefs than true Bayesian updating. This may be framed as recency bias. Notably, the overall recency bias we observed stands in contrast to a well-established tendency for humans to under-update beliefs compared to an ideal Bayesian observer (68) and likely reflects that our prior in this task is in fact just a different modality of evidence that is relatively uninformative (no quantification of the fisherman’s position dock is provided to participants). Intriguingly, this recency bias was not present in those with higher levels of rumination: high rumination was associated with update strength consistent with the ideal Bayesian observer model. One possible interpretation is that rumination, which is typically past-focused and characterized by repeated reprocessing of previously encountered information (52), may bias against incorporation of new evidence as it becomes available. This distinction may also help explain why the effect was specific to rumination rather than worry, which is more future-oriented and often characterized by heightened information seeking under uncertainty. The specificity of the update strength finding to the reflective pondering component of rumination suggests this may be related to a more deliberate style of belief-updating in general. We speculate that the brooding component of rumination—characterized by repetitive focus on negative emotions—would be better captured by a variant of the task incorporating negative emotional stimuli.

Notably, neither worry nor rumination was associated with overall deviation from the ideal Bayesian observer. This is consistent with previous studies in reward-learning that have found overall performance to be intact in individuals with mood and/or anxiety disorders (38, 39) and suggests that individuals with elevated RNT broadly retain the capacity to engage in normative belief-updating when evaluated at a coarse level. It also underscores the importance of decomposing belief-updating into component processes. While the lack of association between RNT and overall belief-updating performance may seem incongruent with the alterations discussed above, there may be some compensation through inverse mechanisms (i.e., greater update strength with greater worry and greater prior weight with greater rumination). However, the task employed in this study occurs in a neutral, non-affective context, which may not be sufficient for revealing overall deficits in belief-updating. There is substantial evidence that affective context and emotional salience can alter how beliefs are revised. For example, experimentally induced mood has been shown to moderate belief-updating (69). Similarly, research on depression and belief updating indicates that affective states and emotional valence shape information integration, with depressed individuals often showing reduced updating in response to positive information and altered weighting of negative information compared to neutral conditions (70, 71). These findings imply that the absence of a strong affective component in “cold” cognitive tasks may mask biases that would emerge under affectively salient or personally relevant belief-updating conditions. Alternatively, the observed alterations in belief-updating coupled with the preserved performance may reflect different strategies that our model is unable to distinguish.

Beyond these dissociable effects of worry and rumination, variability in more basic cognitive capacities may further shape belief updating. Consistent with prior work demonstrating superior Bayesian reasoning among individuals with high working memory span (72), we found that stronger working memory maintenance was associated with more optimal Bayesian performance. This supports the view that maintenance processes enable the stable integration of prior information with incoming evidence to support optimal decision-making, which remains largely intact in individuals with RNT. Notably, however, working memory was not directly associated with the belief updating parameter itself, which may reflect the extent to which updating in this task is supported by more automatic, heuristic processes rather than deliberative, working memory-dependent computation (73). Visuospatial processing also emerged as relevant. In line with computational accounts indicating that anxiety is associated with slower updating at the level of perceptual evidence accumulation rather than decision-making per se (74), better visuospatial perception in our sample was linked to lower update strength–closer to the ideal Bayesian observer and reflecting reduced recency bias. Importantly, this association was independent of the effect of rumination on update strength, suggesting that the belief updating differences associated with RNT in older adults cannot be explained by basic cognitive constraints.

The Fish task proved to be a feasible and well-tolerated paradigm in an older adult sample, with participants demonstrating strong overall agreement with the Bayesian model while also showing enough variability to capture meaningful individual differences. This supports the utility of the task for isolating interpretable belief-updating parameters in later life, a population often underrepresented in computational psychiatry studies. While the Fish task as implemented does not contain a learning component (i.e., no feedback is provided to the participants), belief-updating is a fundamental process in learning behavior. The ability to explicitly quantify specific components of the belief-updating process offers a mechanistic bridge between RNT symptoms and formal models of learning and inference for future studies. One open question is whether these belief-updating processes are implemented with an explicit or implicit strategy and how individual differences in this facet are reflected at the parametric level. Indeed, there is strong evidence for implicit Bayesian updating processes in sensorimotor learning (75, 76), but also in higher order processes (43, 77).

While many studies have used computational approaches to investigate anxiety and depression (30, 31, 78), the remarkable heterogeneity of these disorders may obfuscate actual deficits that are more closely tied to specific symptoms like RNT. This focus on symptoms rather than diagnoses allows for a broader application of the results across disorders and complements similar work focused on reinforcement learning based research on RNT (79). Importantly, the present findings suggest that worry and rumination—although often grouped under the broader RNT umbrella—are supported by distinct computational mechanisms. This raises important conceptual and clinical implications: treating RNT as a unitary process may obscure meaningful differences in the underlying cognitive dynamics that give rise to these symptom expressions. At the same time, the shared classification of worry and rumination within RNT may still be useful at the descriptive level, insofar as both involve repetitive, negatively valenced thought. A more nuanced framework may therefore be warranted, in which RNT is conceptualized as a higher-order construct encompassing related but mechanistically dissociable processes. Consistent with this view, the Fish task allows for dissociation of distinct belief-updating processes that may map onto different therapeutic targets, providing a flexible framework for future behavioral, neuroimaging, and interventional work. The neural encoding warrants future attention as our group has previously shown that many of the key brain regions involved in worry induction (80, 81) are the same regions recruited for belief-updating (dorsolateral prefrontal, insular, posterior parietal, and dorsal anterior cingulate cortices) (82–84). It remains an open question whether shared dysfunction in these regions, a competition for neural resource in these regions, or another explanation altogether underlies these findings.

While this study has enabled a first-of-its-kind investigation of the belief-updating correlates of RNT, it does have several limitations. The cross-sectional design precludes causal inferences about the relationship between belief-updating deficits and RNT. While the dimensional recruitment strategy enhances sensitivity to individual differences, the sample was restricted to older adults, limiting generalizability to younger populations. Future studies should clarify whether these findings are specific to late-life or more general features of RNT across the lifespan. Although the Bayesian model provides a principled framework, it represents an abstraction of real-world belief formation and may not capture all aspects of RNT as it occurs in daily life, in particular the negative valence and self-relevance components of RNT. Future studies would benefit from increasing the ecological validity of the belief-updating task such that it captures these important aspects of RNT. It may also be worthwhile to consider other models of belief-updating and whether participants vary in their use of such strategies. Further, the prior in this Fish task is another type of observation, which may be less “sticky” than deeply held beliefs. The exclusion of participants with poor task understanding, while necessary for model validity, may bias the sample toward higher-functioning individuals.

In summary, this study provides the first evidence that worry and rumination are associated with distinct alterations in belief-updating despite preserved overall performance. These findings support a mechanistic account of repetitive negative thinking as arising from maladaptive weighting of information rather than global cognitive deficits. Ultimately, identifying process-specific mechanisms of RNT may enable more precise, personalized approaches to treatment, particularly in older adults for whom standard interventions are often less effective.

## Data Availability

The raw data supporting the conclusions of this article will be made available by the authors, without undue reservation.
