# Supplementary material for: Gone fishin’… for distinct patterns of belief-updating in late-life worry and rumination
Source: Front Psychiatry. 2026 Jul 15;17:1824838. doi: 10.3389/fpsyt.2026.1824838 (PMC13415504; doi:10.3389/fpsyt.2026.1824838)
Supplement: Supplementary file 1 [file SupplementaryFile1.docx]

**Supplementary Materials**

*Model comparison and assessment of fit*

We evaluated a set of alternative belief-updating models that differed in the number of free parameters governing the weighting of prior beliefs and likelihood information in which $\beta_{1}$ or $\beta_{2}$were fixed to unity using offset terms.

Original model:

$y \sim\beta_{0}+\beta_{1}x_{1}+\beta_{2}x_{2}$ (S1)

Comparison models:

1. $y \sim\beta_{0}+x_{1}+x_{2}$ (S2)

2. $y \sim\beta_{0}+\beta_{1}x_{1}+x_{2}$ (S3)

3. $y \sim\beta_{0}+x_{1}+\beta_{2}x_{2}$ (S4)

Model fit was assessed using Bayesian Information Criterion (BIC). The original model provided the best fit (mean BIC = 32.3 $\pm$ 58.6), outperforming the comparison models (Model 1: 95.2 $\pm$ 30.4; Model 2: 87.3 $\pm$ 29.9; Model 3: 47.5 $\pm$ 67.6).

*Parameter recovery analysis*

To assess the identifiability of model parameters, we conducted a parameter recovery analysis using the best-fitting model. For each participant, we extracted the fitted parameters (intercept $\beta_{0}$, prior weight $\beta_{1}$, likelihood weight $\beta_{2}$) along with the standard deviation of the residuals, which served as an estimate of observation noise. Synthetic datasets were then generated for each participant by simulating responses using their fitted parameters. Behavioral recovery was assessed by comparing simulated and observed responses using Spearman correlations across trials and within participants. To assess parameter recovery, the same model was refit to the simulated data for each participant. Recovered parameters were then compared to the original fitted parameters using Spearman rank correlations across participants. High correlations between true and recovered parameters ($\beta_{1}$ Spearman $\rho$ = 0.95; $\beta_{0}$ Spearman $\rho$ = 0.93; p < 2.2 e-16) indicate that the model parameters are identifiable given the data.

**Supplementary Table 1.**  *Model summary for deviation (i.e., performance)*.

| Parameter | Beta | Std. Error | t | p | VIF |
| --- | --- | --- | --- | --- | --- |
| (Intercept) | 0.04 | 0.04 | 1.13 | 0.26 | - |
| PSWQ | -0.03 | 0.04 | -0.79 | 0.43 | 1.60 |
| RSQ | 0.03 | 0.03 | 0.87 | 0.38 | 1.44 |
| **Age** | **0.08** | **0.03** | **2.57** | **0.01** | 1.13 |
| Sex | -0.11 | 0.07 | -1.65 | 0.10 | 1.16 |
| Education | -0.04 | 0.03 | -1.16 | 0.25 | 1.13 |

**Supplementary Table 2.**  *Model summary for prior weight*.

| Parameter | Beta | Std. Error | t | p | VIF |
| --- | --- | --- | --- | --- | --- |
| (Intercept) | 0.09 | 0.08 | 1.09 | 0.28 | - |
| **PSWQ** | **-0.24** | **0.08** | **-2.89** | **<0.01** | 1.60 |
| RSQ | 0.09 | 0.08 | 1.17 | 0.24 | 1.44 |
| Age | 0.08 | 0.07 | 1.12 | 0.26 | 1.13 |
| Sex | -0.24 | 0.15 | -1.59 | 0.11 | 1.16 |
| **Education** | **-0.17** | **0.07** | **-2.49** | **0.01** | 1.13 |

**Supplementary Table 3.**  *Model summary for update strength*. Significant results bolded. Females are the reference group for sex.

| Parameter | Beta | Std. Error | t | p | VIF |
| --- | --- | --- | --- | --- | --- |
| (Intercept) | -0.08 | 0.14 | -0.56 | 0.57 | - |
| PSWQ | 0.23 | 0.14 | 1.59 | 0.12 | 1.69 |
| **RSQ** | **-0.34** | **0.13** | **-2.55** | **0.01** | 1.47 |
| Age | 0.15 | 0.12 | 1.25 | 0.21 | 1.14 |
| Sex | 0.21 | 0.25 | 0.83 | 0.41 | 1.17 |
| Education | -0.15 | 0.12 | -1.28 | 0.21 | 1.13 |

**Supplementary Table 4.**  *Model summary for update strength with rumination subscales*. Significant results bolded. Females are the reference group for sex.

| Parameter | Beta | Std. Error | t | p | VIF |
| --- | --- | --- | --- | --- | --- |
| (Intercept) | -0.09 | 0.14 | -0.63 | 0.53 | - |
| PSWQ | 0.10 | 0.16 | 0.65 | 0.52 | 2.00 |
| RSQ: Brooding | 0.03 | 0.17 | 0.16 | 0.88 | 2.19 |
| **RSQ: Reflection** | **-0.32** | **0.13** | **-2.47** | **0.02** | **1.38** |
| Age | 0.13 | 0.12 | 1.08 | 0.29 | 1.17 |
| Sex | 0.24 | 0.25 | 0.92 | 0.36 | 1.18 |
| Education | -0.11 | 0.12 | -0.92 | 0.36 | 1.14 |

**Supplementary Table 5.** *Model summary for deviation sensitivity analysis including anxiety and depression symptoms scores.* Significant results bolded. Females are the reference group for sex.

| Parameter | Beta | Std. Error | t | p | VIF |
| --- | --- | --- | --- | --- | --- |
| (Intercept) | 0.04 | 0.04 | 0.97 | 0.33 | - |
| PSWQ | -0.01 | 0.04 | -0.36 | 0.72 | 1.79 |
| RSQ | 0.05 | 0.04 | 1.12 | 0.26 | 2.11 |
| **Age** | **0.09** | **0.03** | **2.72** | **0.01** | 1.15 |
| Sex | -0.09 | 0.07 | -1.32 | 0.19 | 1.27 |
| Education | -0.04 | 0.03 | -1.25 | 0.21 | 1.16 |
| HARS | -0.06 | 0.04 | -1.34 | 0.18 | 1.95 |
| HDRS | 0.02 | 0.04 | 0.46 | 0.65 | 1.73 |

**Supplementary Table 6.** *Model summary for prior strength sensitivity analysis including anxiety and depression symptoms scores.* Significant results bolded. Females are the reference group for sex.

| Parameter | Beta | Std. Error | t | p | VIF |
| --- | --- | --- | --- | --- | --- |
| (Intercept) | 0.08 | 0.09 | 0.94 | 0.35 | - |
| **PSWQ** | **-0.19** | **0.09** | **-2.15** | **0.03** | 1.79 |
| RSQ | 0.15 | 0.10 | 1.52 | 0.13 | 2.12 |
| Age | 0.09 | 0.07 | 1.32 | 0.19 | 1.15 |
| Sex | -0.22 | 0.16 | -1.37 | 0.17 | 1.27 |
| **Education** | **-0.18** | **0.07** | **-2.47** | **0.02** | 1.16 |
| HARS | -0.17 | 0.10 | -1.75 | 0.08 | 1.95 |
| HDRS | 0.03 | 0.09 | 0.38 | 0.70 | 1.73 |

**Supplementary Table 7.** *Model summary for update strength sensitivity analysis including anxiety and depression symptoms scores.* Significant results bolded. Females are the reference group for sex.

| Parameter | Beta | Std. Error | t | p | VIF |
| --- | --- | --- | --- | --- | --- |
| (Intercept) | -0.05 | 0.14 | -0.35 | 0.73 | - |
| PSWQ | 0.18 | 0.15 | 1.16 | 0.25 | 1.87 |
| **RSQ** | **-0.35** | **0.17** | **-2.11** | **0.04** | 2.14 |
| Age | 0.13 | 0.12 | 1.09 | 0.28 | 1.16 |
| Sex | 0.15 | 0.27 | 0.57 | 0.57 | 1.27 |
| Education | -0.14 | 0.12 | -1.14 | 0.26 | 1.16 |
| HARS | 0.20 | 0.16 | 1.23 | 0.22 | 1.95 |
| HDRS | -0.14 | 0.15 | -0.93 | 0.36 | 1.73 |

**Supplementary Table 8.** *Model summary for deviation sensitivity analysis including anxiety and depression diagnoses.* Significant results bolded. Females are the reference group for sex.

| Parameter | Beta | Std. Error | t | p | VIF |
| --- | --- | --- | --- | --- | --- |
| (Intercept) | 0.03 | 0.05 | 0.73 | 0.46 | - |
| PSWQ | -0.03 | 0.04 | -0.78 | 0.44 | 1.73 |
| RSQ | 0.02 | 0.04 | 0.54 | 0.59 | 1.66 |
| **Age** | **0.09** | **0.03** | **2.78** | **0.01** | 1.16 |
| Sex | -0.13 | 0.07 | -1.85 | 0.06 | 1.15 |
| Education | -0.05 | 0.04 | -1.33 | 0.18 | 1.14 |
| GAD | 0.08 | 0.09 | 0.89 | 0.38 | 1.48 |
| MDD | -0.03 | 0.09 | -0.39 | 0.70 | 1.13 |

**Supplementary Table 9.** *Model summary for prior strength sensitivity analysis including anxiety and depression diagnoses.* Significant results bolded. Females are the reference group for sex.

| Parameter | Beta | Std. Error | t | p | VIF |
| --- | --- | --- | --- | --- | --- |
| (Intercept) | 0.02 | 0.10 | 0.18 | 0.86 | - |
| **PSWQ** | **-0.25** | **0.09** | **-2.68** | **0.01** | 1.73 |
| RSQ | 0.03 | 0.09 | 0.37 | 0.71 | 1.66 |
| Age | 0.10 | 0.07 | 1.31 | 0.19 | 1.16 |
| Sex | -0.27 | 0.16 | -1.71 | 0.09 | 1.15 |
| **Education** | **-0.21** | **0.08** | **-2.63** | **0.01** | 1.14 |
| GAD | 0.18 | 0.21 | 0.89 | 0.37 | 1.48 |
| MDD | 0.10 | 0.19 | 0.52 | 0.61 | 1.13 |

**Supplementary Table 10.** *Model summary for update strength sensitivity analysis including anxiety and depression diagnoses.* Significant results bolded. Females are the reference group for sex.

| Parameter | Beta | Std. Error | t | p | VIF |
| --- | --- | --- | --- | --- | --- |
| (Intercept) | -0.19 | 0.16 | -1.17 | 0.25 | - |
| PSWQ | 0.18 | 0.15 | 1.19 | 0.24 | 1.76 |
| **RSQ** | **-0.47** | **0.15** | **-3.24** | **<0.01** | 1.70 |
| Age | 0.21 | 0.12 | 1.84 | 0.07 | 1.18 |
| Sex | 0.14 | 0.25 | 0.57 | 0.57 | 1.15 |
| **Education** | **-0.32** | **0.13** | **-2.57** | **0.01** | 1.14 |
| GAD | 0.54 | 0.33 | 1.64 | 0.11 | 1.44 |
| MDD | -0.21 | 0.30 | -0.69 | 0.49 | 1.12 |


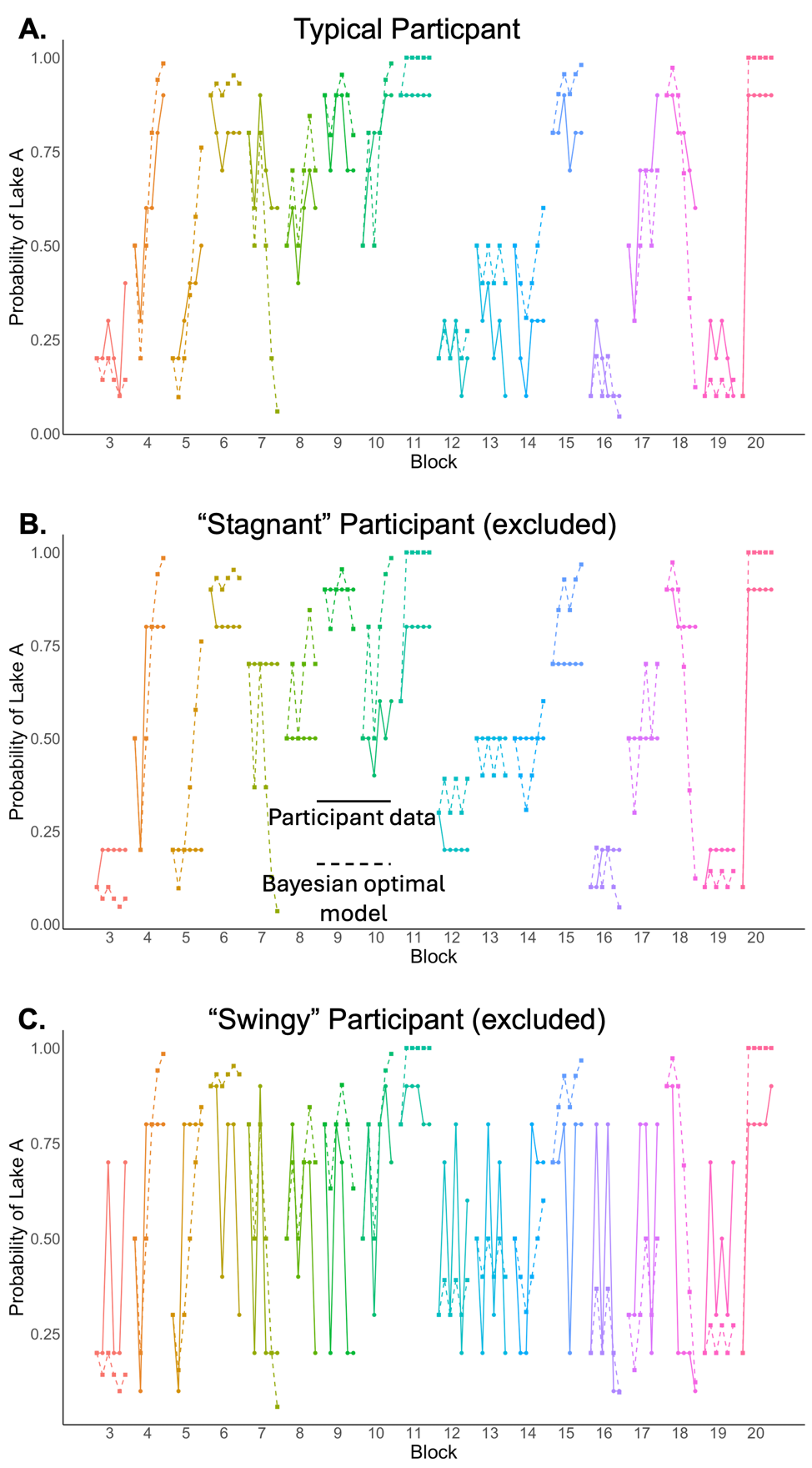


**Supplementary Figure 1.** *Example Participant Data*. Example data is shown for three participants with observed behavior indicated by solid lines and Bayesian optimal behavior indicated by the dashed lines. There are six data points for each block: one for the prior and five for the sequence of fish caught in that block. Note that there is no optimal value for the prior valuation, so the Bayesian optimal model always begins with an initial prior that matches the participant’s value. The block numbering beings at 3 because blocks 1 and 2 are for instruction and training, respectively. **A**. Data from a typical participant shows reasonable overall agreement with the Bayesian optimal model while also containing idiosyncratic deviations that allow for unique quantification of belief-updating. **B**. Data from the median participant excluded for “stagnant” behavior (failure to adjust rating on more than 50 of the 80 trails) suggests a failure to engage in the task more than perseveration-related slowing in updates. **C**. Data from the median participant excluded for “swingy” behavior (switching which lake was rated as more likely at least 50% more than the Bayesian optimal model) suggests participants did not understand that all of the fish from a given block came from the same lake.


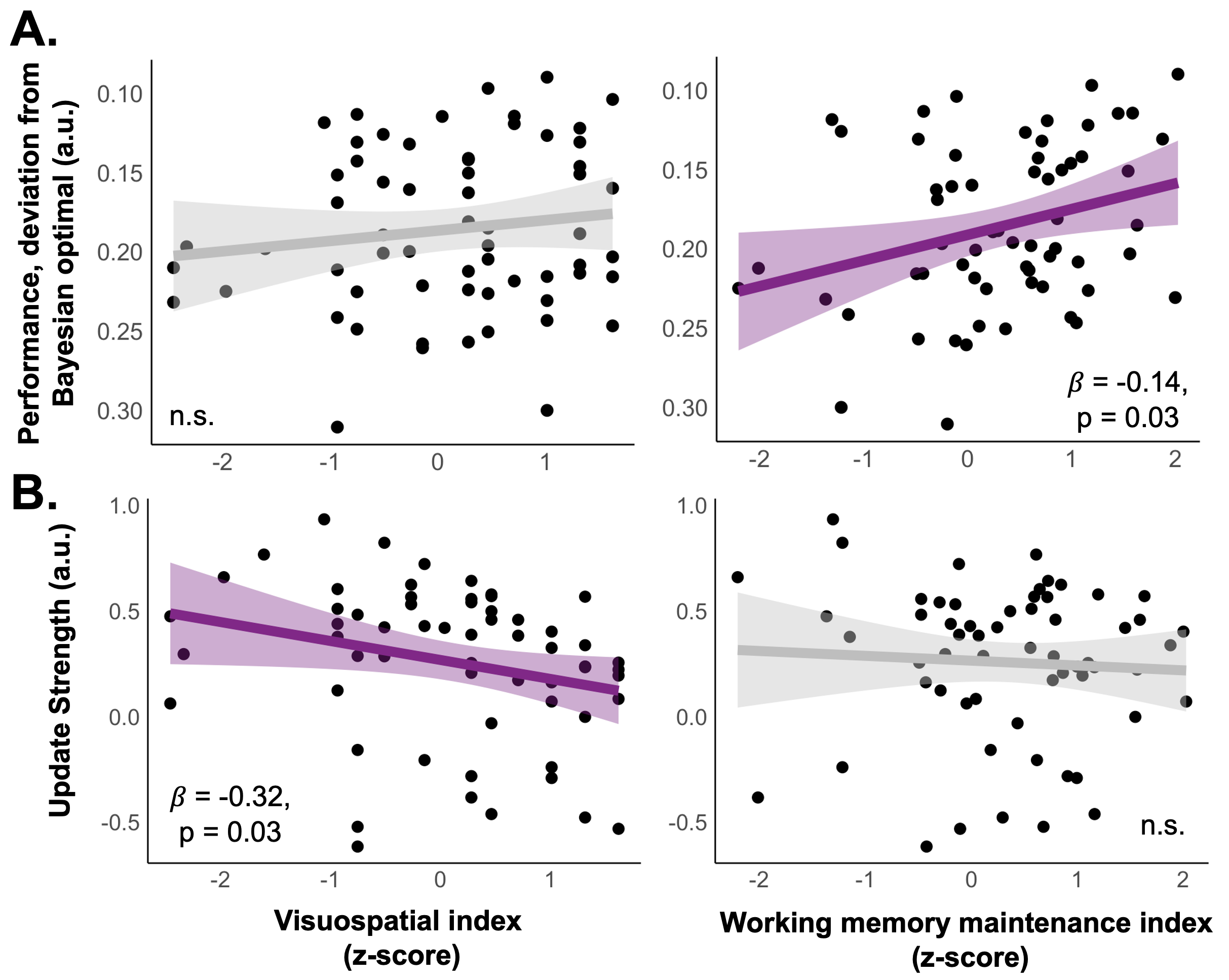


**Supplementary Figure 2.** *Associations Between Model Parameters and Cognitive Indices.* **A**. Greater working memory maintenance was associated with more optimal performance (i.e., smaller deviation from Bayesian optimal model). **B**. Visuospatial processing was negatively associated with update strength. No significant associations were observed between cognitive indices and prior strength, nor between other cognitive domains and performance or update strength.
